# Stener-Like Lesion of the Radial Collateral Ligament of the Proximal Interphalangeal Joint of the Fifth Finger: A Case Report

**DOI:** 10.7759/cureus.70544

**Published:** 2024-09-30

**Authors:** Bruno Cocina, Karim Abdelkafi, Florent Taquet, Sophie Van den Dungen, Katerina Cermak

**Affiliations:** 1 Orthopedic Surgery, Hôpital CHIREC Braine-l'Alleud - Waterloo, Braine-l'Alleud, BEL; 2 Radiology, Hôpital CHIREC Braine-l'Alleud - Waterloo, Braine-l'Alleud, BEL; 3 Orthopedic Surgery, Hôpitaux Iris Sud Bruxelles, Bruxelles, BEL; 4 Hand Surgery, Hôpital CHIREC Braine-l'Alleud - Waterloo, Braine-l'Alleud, BEL

**Keywords:** collateral ligament, finger extensor, hand surgery, open reduction, proximal interphalangeal joint, stener effect, trauma

## Abstract

We present a case of volar subluxation of the fifth finger proximal interphalangeal (PIP) joint following a volleyball trauma. The injury was initially misdiagnosed as a central slip lesion. The persistent pain and PIP joint stuck in flexion led us to perform a second ultrasound exam that showed a complete rupture of the radial collateral ligament and interposition of the radial band of the extensor between the edges of the torn ligament and in the PIP joint. The patient underwent surgery. The radial lateral band was released from the joint space, and the radial collateral ligament was repaired. Among the many cases of PIP joint sprains encountered in busy emergency departments and by hand surgeons in consultation, this insidious presentation, which can easily be missed, should be searched for. If missed, these lesions can lead to very poor outcomes for the patient.

## Introduction

Proximal interphalangeal (PIP) joint sprains are very common during hand surgery consultations. The finger is painful, especially shortly after the traumatic incident, and the range of motion can be limited by the pain. It can be difficult to know when to ask for additional imagery.

The first assessment of a sprain or a subluxated finger is the X-ray. If any doubt persists, an ultrasound should be performed. Among the ultrasound findings of a sprained PIP joint, we must look for a tear of the radial or ulnar ligament, a volar plate disruption, with or without an avulsion of phalanx, or a central slip injury of the extensor.

Very rare cases of interposition of a structure between the edges of a ligament tear or in the PIP joint occur. This type of interposition creates a “Stener-like” effect.

The Stener lesion is well known when considering the lesion of the ulnar collateral ligament of the metacarpophalangeal (MCP) joint of the thumb [1]. An interposition of the adductor pollicis aponeurosis in the ulnar collateral ligament tear prevents the ligament from healing and should prompt surgical intervention and surgical repair.

By extension, any interposition of a tendon’s structure between two parts of a ruptured ligament, hindering its healing, is called a Stener-like effect. It has been described in other sites of the fingers. However, a Stener-like effect of the PIP joint remains very rare, which is why we present the following case.

## Case presentation

A 48-year-old female patient, a recreational volleyball player, presented to the consultation the day after a trauma to her left fifth finger while playing volleyball. She was a left-handed kindergarten teacher. She had previously been treated the year before for a massive ossification removal of the PIP of her left fourth finger, caused by an old volleyball injury.

**Figure 1:**
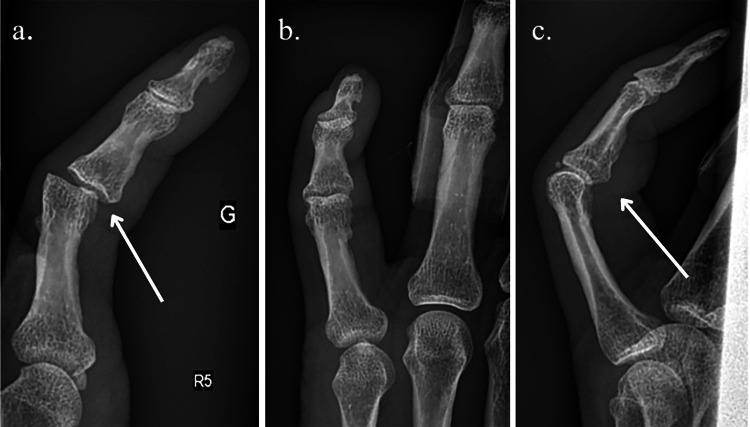
X-rays before and after reduction in the emergency department. (a) Initial X-ray (white arrow: ulnar subluxation of the PIP joint). (b,c) X-rays after reduction ((c) white arrow: flexum of the PIP). PIP, proximal interphalangeal

She now presented with a swollen, painful PIP of her fifth finger. She was treated in the emergency service of another hospital, where a reduction of a subluxation of the PIP joint of her fifth finger was performed (Figure 1).

**Figure 2:**
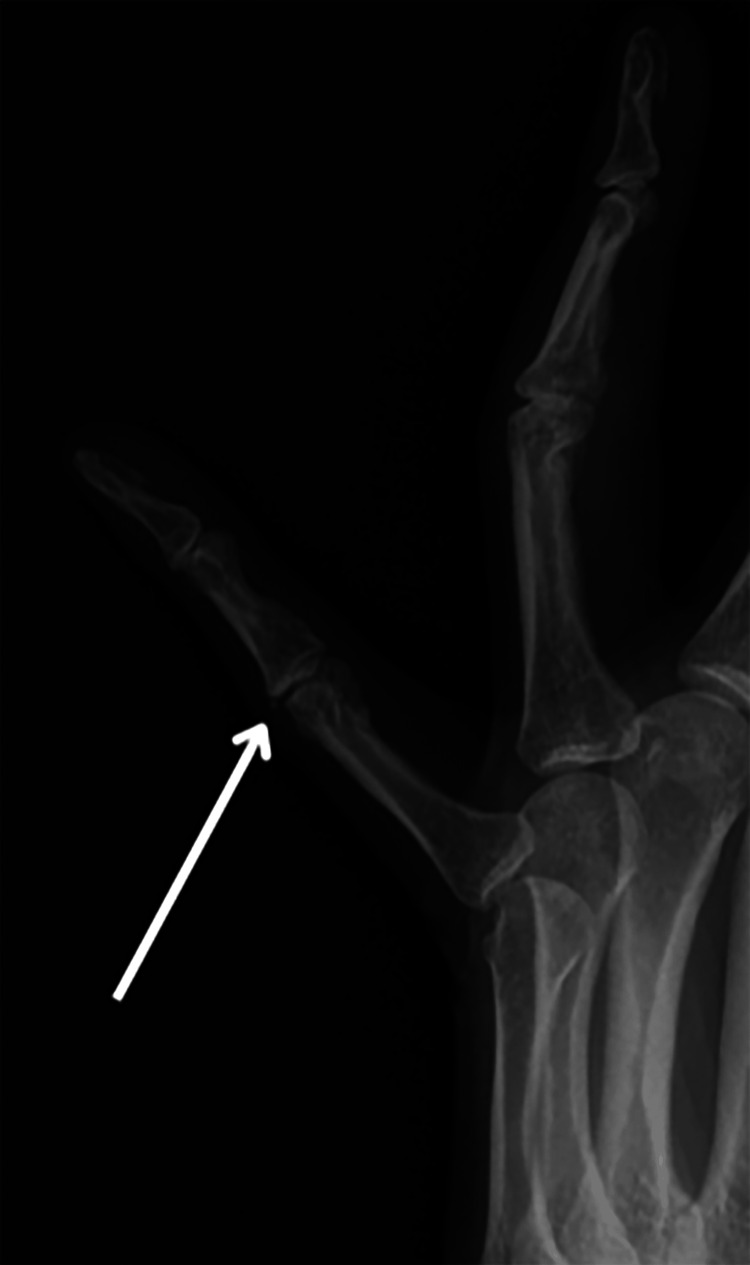
Bone avulsion previously seen on the dorsal aspect of the proximal interphalangeal (PIP) joint one year ago on X-ray (white arrow).

The initial X-rays showed an ulnar subluxation of the PIP joint (Figure 1a), which appears to be resolved after the manipulation (Figure 1b), with a tendency of flexum of the PIP on the profile (Figure 1c). We noted that the micro avulsion seen on the dorsal aspect of the PIP joint was already present on the X-rays of the year before when the patient was treated for the fourth finger (Figure 2).

Her swollen fifth finger was very painful during mobilization. Her flexor tendons were functional, but the extension of the PIP was painful, and a flexum of the PIP joint remains, which was painful when attempting passive and active extension. The first day after a subluxated trauma can be painful, and limitation of the range of motion can occur. However, because of the lack of passive extension, we decided, in this case, to ask for an ultrasound.

The ultrasound was performed by a very experienced osteo-articular radiologist. Swelling of the radial compartment and the hemarthrosis of the PIP joint limited the interpretation of the exam, but the findings showed that the flexor tendons and volar plate were normal. The radial collateral ligament of the PIP joint was not properly seen and was suspected to be injured. A tear of the central slip of the extensor was also suspected.

We then made a PIP splint to treat a central slip tear. It was rarely difficult to extend the finger in the splint, so we asked the patient to come back after a few days to redo the brace, hoping the pain would allow us to properly extend the PIP joint.

She came back after 11 days, still painful, with a 30° flexum of the PIP joint and a persisting inability to actively extend the PIP joint. When passive extension was attempted, the pain was sharp and now irradiated more proximally on the extensor tendon.

**Figure 3:**
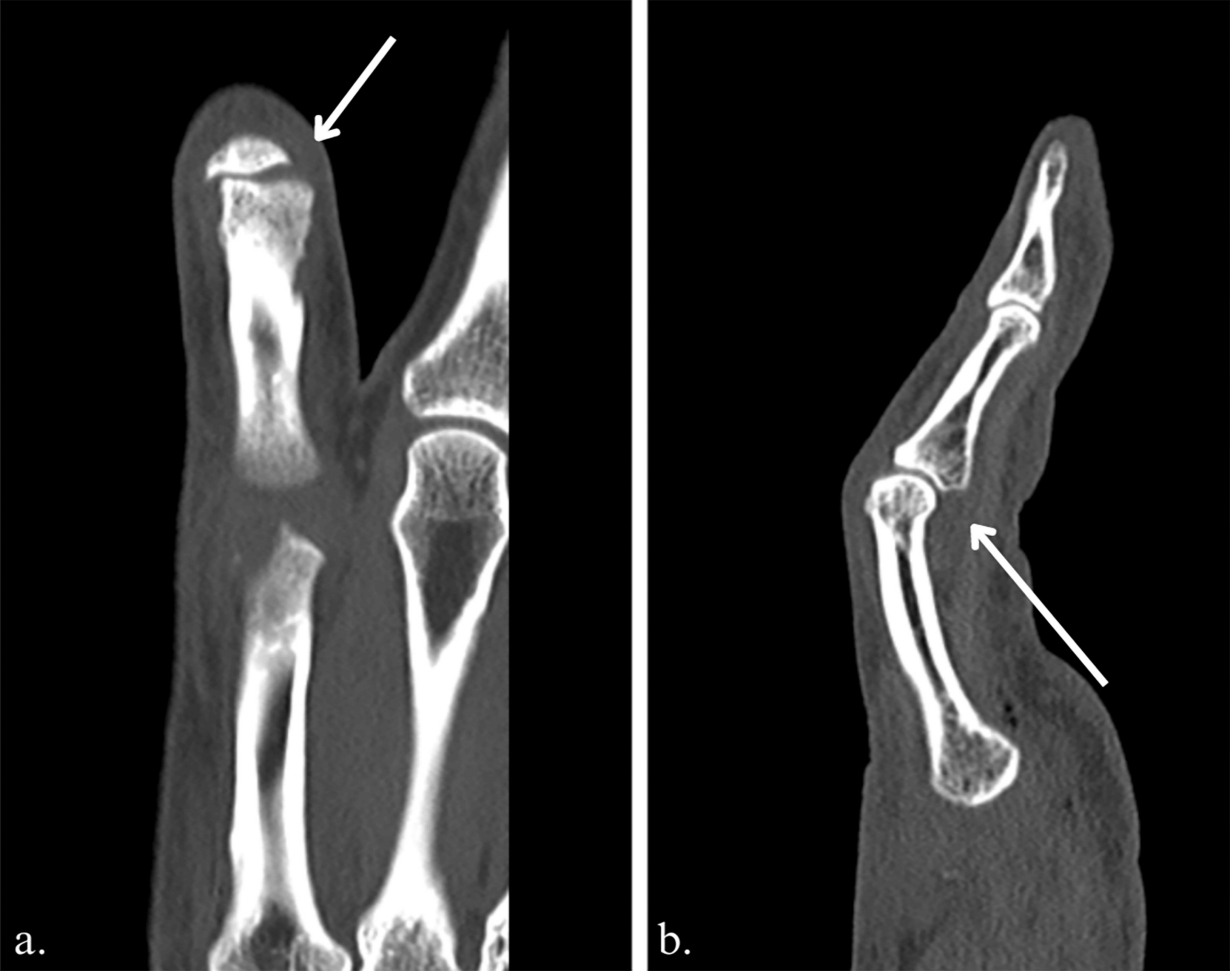
CT scan showing a (a) radial gap of the proximal interphalangeal (PIP) joint (white arrow) suggesting an interposition and on (b) the flexum (white arrow).

We then did a CT scan and a new ultrasound. The CT scan showed a radial gap of the PIP joint, suggesting an interposition (Figure 3).

The new sonography, performed by the same experienced radiologist, showed, this time, a tear-free continuous central band of the extensor. It also confirmed a complete lesion of the radial collateral ligament of the PIP joint and suspected an interposition of the radial collateral band of the extensor mechanism in the PIP joint and between the end of the radial ligament and its attachment to the proximal phalanx, creating a Stener-like effect as well as the interposition of the tendon band in the joint.

**Figure 4:**
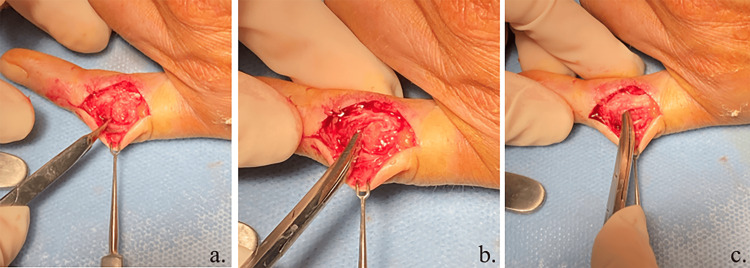
Intraoperative views of the fifth proximal interphalangeal (PIP) joint (a) Unusual aspect of the radial side of the PIP joint showing the surfaces of the bones but no extensor band. (b) An instrument is used to remove the radial band of the extensor blocked in the articulation. (c) The radial band of the extensor is removed from the articulation and is a little bruised but continuous.

The patient was then taken to the operating room. An incision on the radial side of the PIP joint of the fifth finger was made. The radial collateral ligament was exposed but hidden by the radial extensor collateral band, which was indeed interposed between the ends of the ligament (Figures 4a-4b); the extensor band was also found blocked in the PIP joint, forcing the joint into flexion. Once the tendon was removed (Figure 4c), full extension was achievable. The extensor band was bruised but not torn. The central band of the extensor was intact. The radial ligament was reinserted on its proximal insertion through a micro-resorbable JuggerKnot (Zimmer Biomet, Warsaw, IN)-type anchor. The joint was stable after reinsertion, and full extension, as well as full flexion, was possible. The skin was closed, and a temporary analgesic PIP splint was placed and removed two days after allowing and encouraging the patient to move while protected by syndactyly for six weeks.

She had 20 sessions of specialized hand physiotherapy and a nocturnal capener splint for a few months. She returned to work after 10 days and was back on the volleyball field after five months. She is now almost two years from surgery. She has occasional mild pain and swelling in the PIP joint when full flexion is achieved and remains with a 10° flexum.

## Discussion

The Stener-like effect has been described in the literature on different sites of the fingers. Doty et al. [2] published a case of interposition of the abductor aponeurosis in a ruptured radial collateral ligament of the thumb, creating a sort of “reverse skier’s thumb” injury.

A few publications also described Stener-like lesions of the collateral ligament of the MCP joints with interposition of the sagittal band of the extensor mechanism [3-6]. In these cases, attention is paid to the important clinical sign of deviation of abducted fingers unable to adduct [3]. This sign should warn the hand surgeon in consultation for an interposition, as, probably, in our case, a flexed PIP joint unable to extend should warn us about an interposition in the PIP joint.

Few similar cases exist. Two authors described an interposition of the radial collateral ligament in the PIP joint with a similar history of lack of reduction and persistent flexum [7,8]. Sharma et al. described the rotatory subluxation occurring in such cases due to the interposition accompanying the flexum deformity and insisted on the insidious presentation that can easily be missed in a busy emergency department. 

Only one author, Assiotis et al., was found to describe a Stener-like effect in the PIP joint in 2021 [9]. In his case, the history was identical to our case, and the radial collateral of the fifth finger was also implicated in the interposition of the radial band of the extensor. Interestingly, the first diagnosis was also, as in our case, a central slip injury. The attention was also drawn to the persistent pain and inability to passively or actively extend the PIP joint.

Another paper [10] described, in 2022, a Stener-like lesion of the ulnar collateral ligament of the PIP joint of the index finger after a PIP traumatic dislocation. The ulnar lateral band was blocked between the two ends of the ulnar collateral ligament but not interposing in the joint. On clinical examination, the flexion of the PIP joint was limited to a 30° angle, and he presented a lack of extension of 15°.

**Figure 5:**
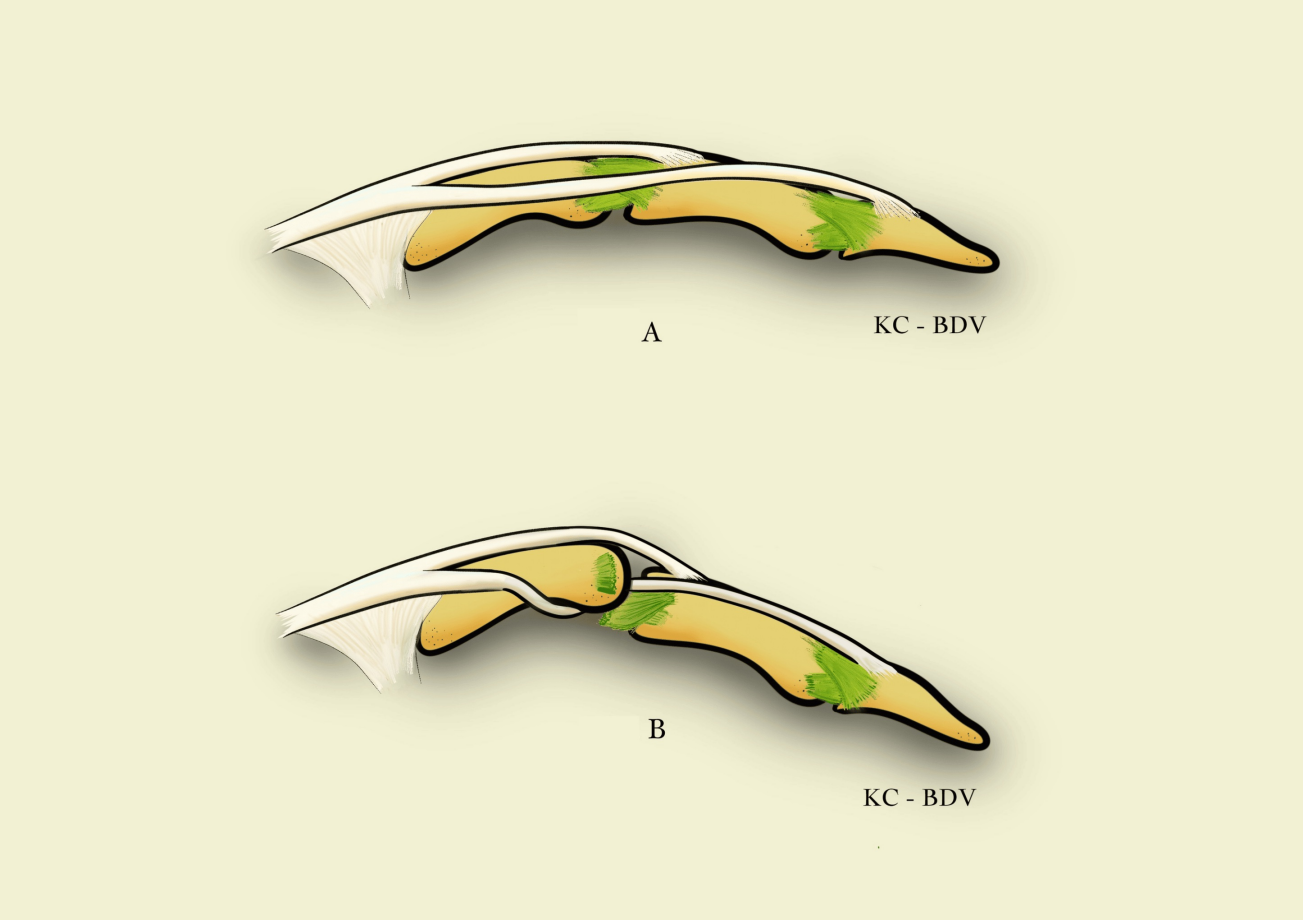
Schematic illustration of the interposition of the extensor band in the proximal interphalangeal (PIP) joint (Stener-like effect). (a) Normal collateral ligament and proper position of the extensor band. (b) Torn collateral ligament, interposition of the extensor band between the edges of the ligament, and incarceration of the tendon in the PIP joint. Drawing by Katerina Cermak and Benoît De Vos.

In the literature and in our case, we found that this type of lesion was difficult to illustrate by intraoperative pictures or imagery assessment. Thus, we made a schematic illustration of the interposition as observed, which is shown in Figure 5.

Care should be taken when attempting to reduce PIP joint dislocations. Persistent flexum after reduction should be a warning, and a closed reduction attempt should not be too aggressive in such cases to prevent damage to the extensor band.

In these rare cases, diagnosis can be made after 11 days, as in our case, or 20 days [10] or even four weeks [9]. In our case, an early ultrasound was made by an experienced radiologist, but swelling and hemarthrosis of the PIP joint limited the visibility and subsequent interpretation of the exam, leading to misdiagnosis. The images were apparently much clearer a few days after the trauma, which teaches us, in addition to looking for this type of lesion, also not to hesitate to redo the ultrasound if clinical evolution is not better.

## Conclusions

As hand surgeons, we treat many sprained PIP joints in our daily practice with variable pain and swelling. X-ray and clinical examination are usually sufficient to deal with these fingers. By reporting this case, we hope to draw attention to the signs of a painful flexed PIP that is blocked and unable to be extended. In these cases, an ultrasound should be asked with specific demand on the interposition of the extensor band. A CT scan can also help to see an asymmetrical gap suggesting interposition. If the initial ultrasound cannot help with the diagnosis because of the hemarthrosis, do not hesitate to redo the exam a few days later and closely monitor those patients. If Stener-like effect is found, surgery is the only option for repairing and ensuring the proper healing of the ligament.
